# Lisdexamfetamine’s Efficacy in Treating Attention Deficit Hyperactivity Disorder (ADHD): A Meta-Analysis and Review

**DOI:** 10.7759/cureus.68324

**Published:** 2024-08-31

**Authors:** Heath Rutledge-Jukes, Pallavi Jonnalagadda, Andrew Paul McIntosh, Saso Krstovski, Nicholas Andriani, Ireland R Smith, Lauren Prendergast, James M Lynch

**Affiliations:** 1 Department of Psychiatry, Washington University School of Medicine, St. Louis, USA; 2 Medical Education, King of the Curve LLC, St. Louis, USA; 3 Department of Medical Education, Washington University in St. Louis School of Medicine, St. Louis, USA; 4 Statistics, King of the Curve LLC, St. Louis, USA; 5 Department of Biological Sciences, University of Michigan, Ann Arbor, USA; 6 Psychiatry, Florida Southern College, Lakeland, USA; 7 Orthopaedics, New York Institute of Technology College of Osteopathic Medicine, Old Westbury, USA; 8 Office of Education and Curriculum, Southern Illinois University School of Medicine, Carbondale, USA; 9 Department of Neurology, University of Colorado Anschutz Medical Campus, Boulder, USA

**Keywords:** hyperactivity, inattentiveness, subscales, meta-analysis, adults, children, adhd-rs-iv, adhd, lisdexamfetamine dimesylate

## Abstract

Lisdexamfetamine dimesylate, a prodrug stimulant, appears to effectively treat Attention-Deficit/Hyperactivity Disorder (ADHD) in children and adults. However, an analysis of the treatment effects of the two subscales (inattentiveness and hyperactivity) within the ADHD Rating Scale-IV (ADHD-RS-IV) has not yet been done to determine if clinical significance may be attributed to either one. Nor has there been a meta-analysis of the individual doses of lisdexamfetamine dimesylate. The current meta-analysis utilizes MEDLINE, Embase, Cochrane, PubMed, and clinicaltrials.gov to identify peer-reviewed studies. Selected studies were eligible if they met the following criteria: English language, randomized-controlled trials, and utilized the ADHD-RS-IV scale to assess the efficacy of lisdexamfetamine on treating ADHD in either children or adults. The primary studies utilized were published between January 2007 and April 2024. Many of these studies calculate effect sizes based on several dosages pooled together rather than by individual dosages. We conducted a random-effects meta-analysis to estimate the effect sizes for these pooled dosages on the full ADHD-RS-IV scale and its subscales, as well as to calculate effect sizes on the same scales based on the individual dosages. Our main outcome measures are the ADHD-RS-IV scale and its subscales in individual doses and pooled results in both children and adults. Adverse events during treatment were also analyzed based on stratified dosages. Eleven publications met our inclusion criteria. The analyses indicate that compared to placebo, lisdexamfetamine effectively alleviates the symptoms outlined by the ADHD-RS-IV. Moreover, there are no differences in the individual subscales or in the safety profile. Lisdexamfetamine demonstrates efficacy in treating the symptoms of ADHD, but we report that differing dosages did not yield significant differences in ADHD symptom management.

## Introduction and background

Classified as a neurodevelopmental disorder, Attention-Deficit/Hyperactivity Disorder (ADHD) can present with an array of symptoms such as inattentiveness, impulsiveness, and hyperactivity [1]. ADHD is estimated to occur in 5.6% to 13.3% of the population [2]. With decreased activation of the prefrontal cortex, people diagnosed with ADHD struggle with inhibition, impulsivity, memory, and planning. People with ADHD have fewer dopamine receptors within the reward pathway, which leads to the symptoms exhibited by this disorder [3,4].

Lisdexamfetamine dimesylate (LDX), a long-acting stimulant prodrug, demonstrates efficacy in alleviating ADHD symptoms in children, adolescents, and adults [3,4]. In contrast to other stimulant drugs that produce long-acting effects, prodrugs are less likely to be abused because they are almost entirely inert until chemically or enzymatically acted upon to release the active moiety drug component [5]. The mechanism for the activation of LDX is nebulous. The active drug undergoes first-pass metabolism, most likely through human peptide transporter 1 (PEPT1) [6]. Erythrocytes enzymatically hydrolyze LDX in a high-capacity system into inactive l-lysine and d-amphetamine, the active stimulant [6,7]. Subsequently, d-amphetamine crosses the blood-brain barrier to exert therapeutic effects by inducing the release of and preventing the reuptake of norepinephrine and dopamine [5-7]. The conversion of LDX to d-amphetamine has the benefit of eliciting a longer duration of action than other amphetamine formulations. Following ingestion, LDX exhibits therapeutic effects for up to 13 hours in children and up to 14 hours in adults [5,7].

ADHD can be measured and categorized using the ADHD Rating Scale-IV (ADHD RS-IV). The scale, inspired by DSM-IV criteria, consists of 18 items that measure inattentiveness and hyperactivity [8]. This scale is widely prevalent in the diagnosis of ADHD symptomatology and has been demonstrated to have high inter-rater reliability, internal consistency, test-retest reliability, convergent and divergent validity, discriminant validity, and responsiveness [9,10]. A parent or a guardian can score a home version of the ADHD RS-IV, and an instructor can score a version given in the school setting. The ADHD RS-IV may be self-reported by adults, while versions for children and adolescents are scored by a parent, guardian, teacher, or clinician, leading to issues when comparing adults and children as inter-rater reliability may skew data [9-11].

Prior studies involving LDX suggest that, while effective at treating ADHD, there are certain dosages that are better in the treatment. Two studies noted that 70 mg yielded more favorable results than 30 mg [12,13]. While previous studies on LDX have considered optimal dose treatments and other factors such as cost analysis, there have been none to our knowledge that have split the individual dosages and analyzed both safety and efficacy. Some questions are apparent. Does LDX have differing efficacies for children, adolescents, and adults? Can effectiveness be improved with consideration of the prominent subscale measures in an individual? Does efficacy vary between races? Should doses be adjusted in regard to gender?

This meta-analysis investigated the impact of child/adolescent and adult LDX dosages on total ADHD-RS-IV, along with LDX's effects on inattention and the hyperactivity/impulsivity subscales [14]. Our study also considered the difference between pooled data and individual dosages. No previous meta-analysis has investigated the efficacy and safety of LDX based on its varying dosages and each of the respective subscales at once; such an investigation could assist prescription protocols for individuals who present specific symptoms (inattention primarily or hyperactivity/impulsivity primarily). Furthermore, we analyze the various optimal dosages of lisdexamfetamine and compare them to one another in both children and adults.

## Review

Methods

Protocol and Selection of Studies for Meta-Analysis

We used the Preferred Reporting Items for Systematic Reviews and Meta-Analyses (PRISMA) methodology. The search was conducted through April 2024 in Medline Complete, PubMed, Embase, Cochrane Library, and ClinicalTrials.gov utilizing the search strategy: (lisdexamfetamine) AND (ADHD).

Inclusion criteria included 1) ADHD treated with LDX, 2) a randomized controlled trial (RCT) with a placebo, 3) English text and, 4) ADHD-RS-IV changes from baseline were used to evaluate efficacy. The exclusion criteria were 1) the study did not record the change in ADHD-RS-IV score, 2) treatment did not include LDX, 3) case studies and, 4) animals were involved.

Literature and Data Extraction

We obtained 1362 references from five databases, and 592 duplicates were removed. The remaining articles were screened by title and abstract, at which point data extraction was performed and assessed based on condition, treatment mode, data availability, and ADHD-RS-IV use. The study population (mean age, sex percentage, dosage) and outcomes were extracted from 11 articles in a full-text review [12,13,15-24].

Outcome Measures

We used both the ADHD-RS-IV scale and its inattentiveness and hyperactivity/impulsivity subscales as primary outcome measures. Adverse events were also reported by percentage for each event.

Quality Assessment

We performed an eight-question quality assessment based on the Grading of Recommendations Assessment, Development and Evaluation (GRADE) assessment for each study included in the analysis [25]. See Table 1. Overall, we only considered two studies to have a potential "high bias" due to their attrition rate exceeding 20% [21,23].

**Table 1:** GRADE Quality Assessment References [12,13,15-21,23,24] GRADE=Grading of Recommendations Assessment, Development and Evaluation; Q1=Random sequence generation; Q2= Allocation concealment; Q3= Blinding of participants and personnel; Q4= Attrition Bias; Q5= Outcome assessment was objective; Q6= Incomplete outcome data; Q7=Selective reporting; Q8= Other bias; U= Unclear; L= Low; H= High

Study/Year	Q1	Q2	Q3	Q4	Q5	Q6	Q7	Q8
Adler et al. (2008) [12] (NCT00334880)	L	L	L	L	L	L	L	L
Adler et al. (2013) [20] (NCT01101022)	L	L	L	U	L	L	L	L
Biederman et al. (2007) [13]	L	L	L	U	L	L	L	L
Biederman et al. (2012) [19]	L	L	L	L	L	L	U	L
Coghill et al. (2013) [16] (NCT00763971)	L	L	L	L	L	L	L	L
Findling et al. (2011) [15]	L	L	L	L	L	L	L	L
Ichikawa et al. (2020) [18]	L	L	L	L	L	L	L	L
Newcorn et al. (2017) [17] (NCT01552902)	L	L	L	L	L	L	L	L
Weisler et al. (2014) [24]	L	L	L	L	L	L	L	L
NCT03260205 [21]	L	L	L	H	L	L	L	L
Wigal et al. (2010) [23] (NCT00697515)	L	L	L	H	L	L	L	L

Meta-Analysis

We performed a random-effects meta-analysis on the ADHD-RS-IV scale and its respective subscales (inattentiveness and hyperactivity/impulsivity). The analysis was stratified based on sample type (adult vs. child) and dosage. We chose to use the random-effects meta-analysis to consider the heterogeneity present among the different clinical trials (a more conservative approach). The adverse events were analyzed utilizing a proportionality forest plot and stratified based on participant age group (adult vs. child). Analyses were conducted through Stata with packages Metan, Metafunnel, and Metaprop.

Results

Study Characteristics

**Figure 1:**
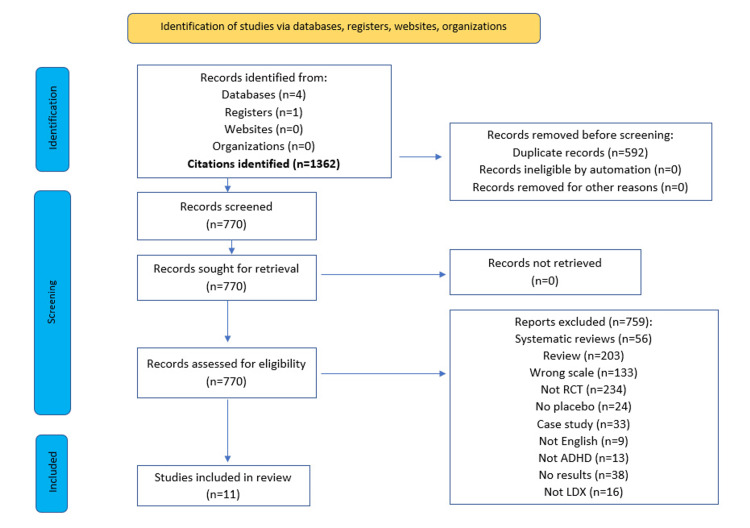
PRISMA flow diagram PRISMA: Preferred Reporting Items for Systematic Reviews and Meta-Analyses

Eleven studies were included based on the PRISMA methodology and search criteria (Figure 1).

All 11 studies were randomized, double-blinded, placebo-controlled trials (Tables 2, 3). The total sample size for the studies was 2732 participants. For LDX, the sample size utilized for the analysis was 1966 participants, with 1169 in the child studies and 797 in the adult studies. For the placebo groups (766 total participants), 526 participants were included in the child studies and 240 in the adult studies.

**Table 2:** Characteristics of included studies (adults) ADHD-RS=Attention Deficit/Hyperactivity Disorder Rating Scale, LDX = lisdexamfetamine dimesylate ^1^This study was a forced-dose escalation in which both 50 mg and 70 mg cohorts were increased from 30 mg [12]. ^2^This is a crossover study with a 4-week dose optimization phase followed by a 2-week crossover phase [23].

Study	ADHD-RS Scale					LDX	Participants		Gender			
Author (Year)	Baseline: Placebo (Mean+SD)	Baseline: LDX (Mean+SD)	Endpoint: Placebo (Mean+SD)	Endpoint: LDX	Change:	Dosage	Placebo	LDX	Male	Female	Age (Mean +SD	Weeks
^1^Adler et al., (2008) [12]	-	40.7 ± 6.3 (30 mg)	-	-	16.2±1.06 (30 mg)	30 mg	62	119	228	192	35.3±10.1 (30 mg)	4 weeks
	-	40.8 ± 7.3 (50 mg)	-	-	17.4±1.05 (50 mg)	50 mg	-	117	-	-	34.2±10 (50 mg)	
	-	41.1 ± 6.0 (70 mg)	-	-	-18.6±1.03 (70 mg)	70 mg	-	122	-	-	35.8±10.5 (70 mg)	
	39.4 ±6.4 (placebo)	-	-	-	-8.2±1.43 (placebo)	-	-	-	-	-	35.2±10.9 (placebo)	
Adler et al., (2013) [20]	-	-	-	-	-10.3±1.35 (LDX)	pooled	80	79	83	76	34.2±10.58 (LDX)	10 weeks
	-	-	-	-	-10.3±1.38 (placebo)	-	-	-	-	-	34.9±11.02 (placebo)	
	-	-	-	-	-	-	-	-	-	-	34.06±10.7 (total)	
Biederman et al., 2012 [19]	30.4 ± 6.7	31.6 ± 7.9 (LDX)	-	-	-18.4±12.6 (LDX)	pooled	34	35	-	-	-	6 weeks
	-	-	-	-	-5.4±9.9 (placebo)	-	-	-	-	-	-	
^2^Wigal et al., 2010 [23]	-	-	-	-	-21.4 ± 7.31 (LDX)	pooled	64	142	88	54	30.5±10.7	6 weeks
	-	-	-	-	-8.7 ± 4	-	-	-	-	-	-	

**Table 3:** Characteristics of included studies (children) ADHD-RS=Attention deficit/hyperactivity disorder rating scale, LDX=Lisdexamfetamine dimesylate, plac=placebo. ^1^This was a forced dose escalation.

Study	ADHD-RS Scale					LDX	Participants		Gender		Age (Mean+SD)	Period
Author (Year)	Baseline: Placebo (Mean+SD)	Baseline: LDX (Mean+SD)	Endpoint: Placebo (Mean+SD)	Endpoint: LDX	Change:	Dose	Placebo	LDX	M	F		Weeks
^1^Biederman et al., 2007 [12]	-	-	-	-	-21.8 (30 mg)	30 mg	72	71	201	89	9.0±1.9 (30 mg)	4 weeks
	-	-	-	-	-23.4 (50 mg)	50 mg	-	74	-	-	8.9±1.8 (50 mg)	
	-	-	-	-	-26.7±1.54 (70 mg)	70 mg	-	73	-	-	8.7±1.8 (70 mg)	
	-	-	-	-	-6.2±1.56 (plac)	-	-	-	-	-	9.4±1.7 (plac)	
Coghill et al., 2013 [22]	-	-	-	-	-24.3±1.16 (LDX)	pooled	110	111	268	64	10.9±2.87 LDX)	7 weeks
	-	-	-	-	-5.7±1.13 (plac)	-	-	-	-	-	11.0±2.82 (plac)	
	-	-	-	-		-	-	-	-	-	10.9±2.77 (total)	
Findling et al., 2011 [23]	-	-	-	-	-18.3±1.25 (30 mg)	30 mg	79	78	220	94	14.6±1.39 (30 mg)	4 weeks
	-	-	-	-	-21.1±1.28 (50 mg)	50 mg	-	79	-	-	14.7±1..29 (50 mg)	
	-	-	-	-	-20.7±1.25 (70 mg)	70 mg	-	78	-	-	14.4±1.31 (70 mg)	
	-	-	-	-	-12.8±1.25 (plac)	-	-	-	-	-	14.5±1.25 (plac)	
	-	-	-	-	Inattention subscale	-	-	-	-	-	-	
Ichikawa et al., 2020 [18]	-	38.1 ± 6.7 (30 mg)	-	-16.38±2.2 (30 mg)	-8.26±1.23 (30 mg)	30 mg	19	19	63	13	10.1±3.1 (30 mg)	4 weeks
	-	37.1 ± 6.9 (50 mg)	-	-18.1±2.35 (50 mg)	-11.23±1.28 (50 mg)	50 mg	-	18	-	-	10.0±2.9 (50 mg)	
	-	37.2 ± 7.8 (70 mg)	-	-16.47±2.3 (70 mg)	-9.62±1.27 (70 mg)	70 mg	-	20	-	-	10.1±2.5 (70 mg)	
	37.9 ± 7.4 (plac)	-	-2.78±2.25 (plac)	-	-0.57±1.25 (plac)	-	-	-	-	-	9.9±2.7 (plac)	
	-	-	-	-	Hyperact/Imp Subscale	-	-	-	-	-	-	
	-	-	-	-	-8.14±1.21 (30 mg)	-	-	-	-	-	-	
	-	-	-	-	-7.02±1.27 (50 mg)	-	-	-	-	-	-	
	-	-	-	-	-7.1±1.26 (70 mg)	-	-	-	-	-	-	
	-	-	-	-	-1.89±1.21 (plac)	-	-	-	-	-	-	
Newcorn et al., 2017 [21]	-	37.2 ± 6.46 (LDX)	-	-	-25.4±0.74 (LDX)	pooled	110	218	61	186	14.6±1.4 (LDX)	4 weeks
	36.1 ± 5.91 (plac)	-	-	-	-17±1.03 (plac)	-	-	-	-	-	14.7±1.4 (plac)	
	-	36.9 ± 6.34 (total)	-	-	-	-	-	-	-	-	14.7±1.4 (total)	
Shire et al., 2015 NCT01552902 [16]	-	-	-	-	-25.6±0.82 (LDX)	pooled	91	184	305	154	14.7+1.4 (LDX)	8 weeks
	-	-	-	-	13.4±1.19 (plac)	-	-	-	-	-	14.8±1.4 (plac)	
	-	-	-	-	-	-	-	-	-	-	14.7±1.4 (total)	
Shire et al., 2020 NCT03260205 [17]	-	-	-	-	-14.2±1.57 (LDX)	5 mg	45	39 (5 mg)	129	62	-	6 weeks
	-	-	-	-	-	10 mg	-	35 (10 mg)	-	-	-	
	-	-	-	-	-9.1±2.36 (plac)	-	-	-	-	-	-	

Treatment-emergent adverse effects were compiled for both adults and children by frequency of event (Tables 4, 5).

**Table 4:** Treatment-emergent adverse events (TEAE) (adults) References [12,20]

	Adler et al.					
	2008 [12]				2013 [20]	
	pla	LDX			pla	LDX
Number (%)		30 mg	50 mg	70 mg		PO
Participant	62	119	117	122	79	80
Any Event	36 (58)	90 (76)	90 (77)	102 (84)	41 (51)	62 (78)
Anorexia	0	4 (3)	8 (7)	6 (5)	0	4 (5)
Anxiety	0	5 (4)	7 (6)	9 (7)	0	4 (5)
↓ Appetite	1 (2)	34 (29)	33 (28)	28 (23)	5 (6)	26 (33)
Diarrhea	0	8 (7)	12 (10)	4 (3)	2 (2)	6 (8)
Dry Mouth	2 (3)	25 (21)	29 (25)	38 (31)	6 (8)	25 (32)
Jittery	0	2 (2)	4 (3)	9 (7)	0	10 (13)
Insomnia	3 (5)	23 (19)	20 (17)	26 (21)	3 (4)	10 (13)
Nausea	0	10 (8)	7 (6)	8 (7)	5 (6)	2 (3)
Irritability	-	-	-	-	3 (4)	8 (10)
Fatigue	-	-	-	-	3 (4)	6 (8)
NPG	-	-	-	-	4 (5)	4 (5)
URI	-	-	-	-	1 (1)	5 (6)
Heart Rate ↑	-	-	-	-	2 (2)	4 (5)
Weight ↓	-	-	-	-	0	8 (10)
Headache	-	-	-	-	2 (2)	20 (25)
Initial Insomnia	-	-	-	-	5 (6)	8 (10)
Libido ↓	-	-	-	-	0	4 (5)
Hyperhidrosis	-	-	-	-	0	4 (5)

**Table 5:** Treatment-emergent adverse events (children) References [12,16-18,22-24]

	Biederman et al.				Coghill et al.		Findling et al.				Ichikawa et al.				NCT01552902		NCT01552915		NCT03260205				
	2007 [12]				2013 [22]		2011 [23]				2020 [18]				2017 [16]		2014 [24]		2020 [17]				
	pla	LDX			pla	LDX	pla	LDX			pla	LDX			pla	LDX	pla	LDX	pla	LDX			
Number (%)		30 mg	50 mg	70mg		PO		30 mg	50 mg	70 mg		30 mg	50 mg	70 mg		70 mg		PO		5 mg	10 mg	20 mg	30 mg
Participant	72	71	74	73	110	111	77	78	77	78	19	19	18	20	110	218	91	184	45	39	35	34	38
Any Event	34 (47)	51 (72)	50 (68)	61 (84)	63 (57)	80 (72)	38 (49)	51 (65)	53 (69)	56 (72)	8 (42)	13 (68)	18 (100)	14 (70)	32 (29)	113 (52)	39 (43)	139 (76)	4 (9)	8 (21)	11 (31)	14 (41)	18 (47)
Anorexia	-	-	-	-	2 (2)	12 (11)	-	-	-	-	-	-	-	-	-	-	-	-	-	-	-	-	-
Anxiety	-	-	-	-	-	-	-	-	-	-	-	-	-	-	-	-	-	-	-	-	-	-	-
↓ Appetite	3 (4)	26 (37)	23 (31)	36 (2)	3 (3)	28 (25)	2 (3)	29 (37)	21 (27)	29 (37)	0	9 (47)	14 (78)	11 (55)	11 (10)	69 (32)	7 (8)	98 (53)	4 (9)	3 (8)	3 (9)	6 (18)	8 (21)
Diarrhea	-	-	-	-	3 (3)	5 (4)	-	-	-	-	-	-	-	-	-	-	-	-	-	-	-	-	-
Dry Mouth	0	2 (3)	2 (3)	6 (8)	-	-	1 (1)	0	6 (8)	4 (5)	-	-	-	-	-	-	1 (1)	15 (8)	0	2 (5)	0	0	0
Jittery	-	-	-	-	-	-	-	-	-	-	-	-	-	-	-	-	-	-	-	-	-	-	-
Insomnia	2 (3)	11 (16)	12 (16)	18 (25)	0	16 (14)	3 (4)	7 (9)	8 (10)	11 (14)	0	0	3 (17)	1 (5)	3 (3)	17 (8)	0	17 (9)	1 (2)	0	1 (3)	0	2 (5)
Nausea	2 (3)	3 (4)	2 (3)	8 (11)	3 (3)	12 (11)	2 (3)	1 (1)	3 (4)	5 (6)	0	0	1 (6)	2 (10)	3 (3)	11 (5)	4 (4)	14 (8) -	-	-			
Irritability	0	8 (11)	6 (8)	7 (10)	0	4 (4)	3 (4)	6 (8)	2 (3)	8 (10)	-	-	-	-	7 (6)	11 (5)	9 (10)	37 (20)	0	3 (8)	4 (11)	3 (9)	4 (10)
Fatigue	-	-	-	-	3 (3)	5 (5)	2 (3)	4 (5)	2 (3)	4 (5)	-	-	-	-	3 (3)	10 (5)	-	-	-	-	-	-	-
NPG	4 (5)	4 (5)	3 (4)	4 (5)	8 (7)	8 (7)	1 (1)	2 (2)	4 (5)	1 (1)	1 (5)	4 (21)	2 (10)	4 (22)	-	-	1 (1)	11 (6)	0	1 (3)	0	2 (6)	2 (5)
URI	-	-	-	-	2 (2)	3 (3)	6 (8)	2 (3)	4 (5)	4 (5)	-	-	-	-	-	-	8 (9)	9 (5)	1 (2)	2 (5)	2 (6)	0	0
Heart Rate ↑	-	-	-	-	-	-	-	-	-	-	-	-	-	-	-	-	0	8 (4)	-	-	-	-	-
Weight ↓	1 (1)	4 (5)	2 (3)	14 (19)	0	15 (14)	0	3 (4)	7 (9)	12 (15)	0	1 (5)	2 (11)	1 (5)	0	23 (10)	1 (1)	37 (20)	0	0	0	2 (6)	0
Headache	7 (10)	7 (10)	7 (10)	12 (16)	22 (20)	16 (14)	10 (13)	9 ( 12)	13 (17)	12 (15)	0	2 (10)	7 (39)	1 (5)	9 (8)	33 (15)	7 (8)	28 (15)	0	0	1 (3)	2 (6)	0
Initial Insomnia	-	-	-	-	1 (1)	4 (4)	-	-	-	-	0	2 (10)	5 (29)	5 (25)	-	-	2 (2)	15 (8)	2 (4)	1 (3)	1 (3)	0	3 (8)
Libido ↓	-	-	-	-	-	-	-	-	-	-	-	-	-	-	-	-	-	-	-	-	-	-	-
Hyperhidrosis	-	-	-	-	-	-	-	-	-	-	-	-	-	-	-	-	-	-	-	-	-	-	-
Upper Abdominal Pain	4 (6)	10 (14)	5 (7)	11 (15)	6 (5)	8 (7)	-	-	-	-	-	-	-	-	2 (2)	11 (5)	4 (4)	12 (7)	-	-	-	-	-
Vomiting	3 (4)	5 (7)	4 (5)	10 (14)	1 (1)	4 (4)	4 (5)	0	1 (1)	2 (3)	-	-	-	-	-	-	-	-	-	-	-	-	-
Dizziness	0	5 (7)	4 (5)	2 (3)	1 (1)	4 (4)	3 (4)	1 (1)	4 (5)	5 (6)	-	-	-	-	0	12 (5)	1 (1)	12 (7)	-	-	-	-	-
Cough	4 (6)	2 (3)	1 (1)	0	0	3 (3)	-	-	-	-	-	-	-	-	-	-	-	-	1 (2)	1 (3)	2 (6)	2 (6)	2 (5)
Nasal Congestion	4 (6)	3 (4)	0	0	-	-	1 (1)	1 (1)	0	5 (6)	-	-	-	-	-	-	-	-	-	-	-	-	-
Constipation	-	-	-	-	-	-	-	-	-	-	-	-	-	-	-	-	-	-	1 (2)	0	0	2 (6)	0

The statistical measures considered are effect size with confidence interval and I^2^. Effect size (ES) indicates the strength of the relationship between two variables-with a larger number indicative of a stronger relationship. In this analysis, the difference between groups is most important. The confidence interval (CI) estimates the precision of the relationship in the groups of interest to provide a plausible range of effect size with wider confidence intervals suggesting less dependable estimates. The heterogeneity of the studies was assessed with the index I^2^. This represents the total variation in heterogeneity to assess the inconsistency among study participants. Values range from 0-100%, with 0 implying no heterogeneity.

ADHD-RS-IV Total

**Figure 2:**
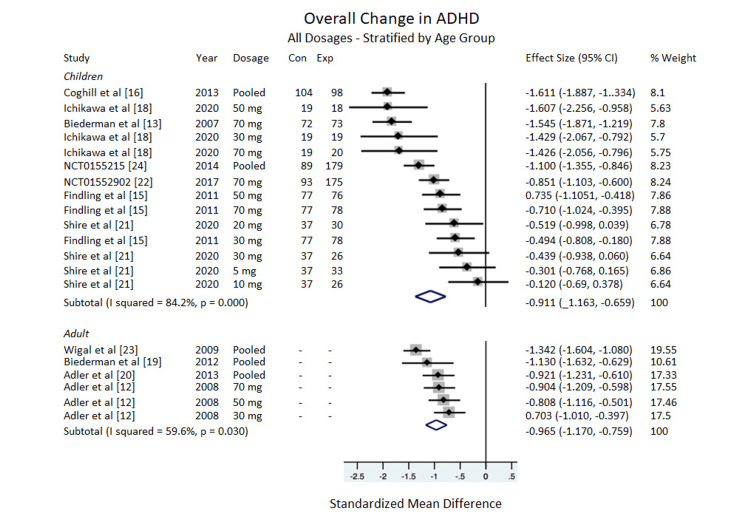
Overall change in ADHD-RS-IV All dosages stratified by age group. ADHD-RS=Attention Deficit/Hyperactivity Disorder Rating Scale, Con=Control group sample size, Exp=Experimental group sample size.

Overall: Compared to the placebo, LDX showed significant efficacy in treating ADHD for adults, ES= -0.965, 95% CI = -1.170 to -0.759, I^2^ = 59.6%. The data for the children subgroup showed a significant improvement in the ADHD-RS-IV scale, ES = - 0.911, 95% CI = -1.163 to -0.659, I^2^ = 84.2% (Figure 2).

Dosages

In adults: Only one adult study included non-pooled results from individual dosages and their impacts on the ADHD-RS-IV scale [12]. The other three studies reported pooled data only (the authors of this meta-analysis were unable to access the data from these studies at an individual dosage level) [19,20,23]. Based on the one study that reported individual dosages, there was no significant difference between the changes in the overall ADHD-RS-IV scale for adults based on dosage. For the 30 mg dosage, ES = -0.703, 95% CI = -1.010 to -0.397. For the 50 mg dosage, -0.808, 95% CI = -1.116 to -0.501. For the 70 mg dosage, ES = -0.904, 95% CI = -1.209 to -0.598 (Figure 2).

**Figure 3:**
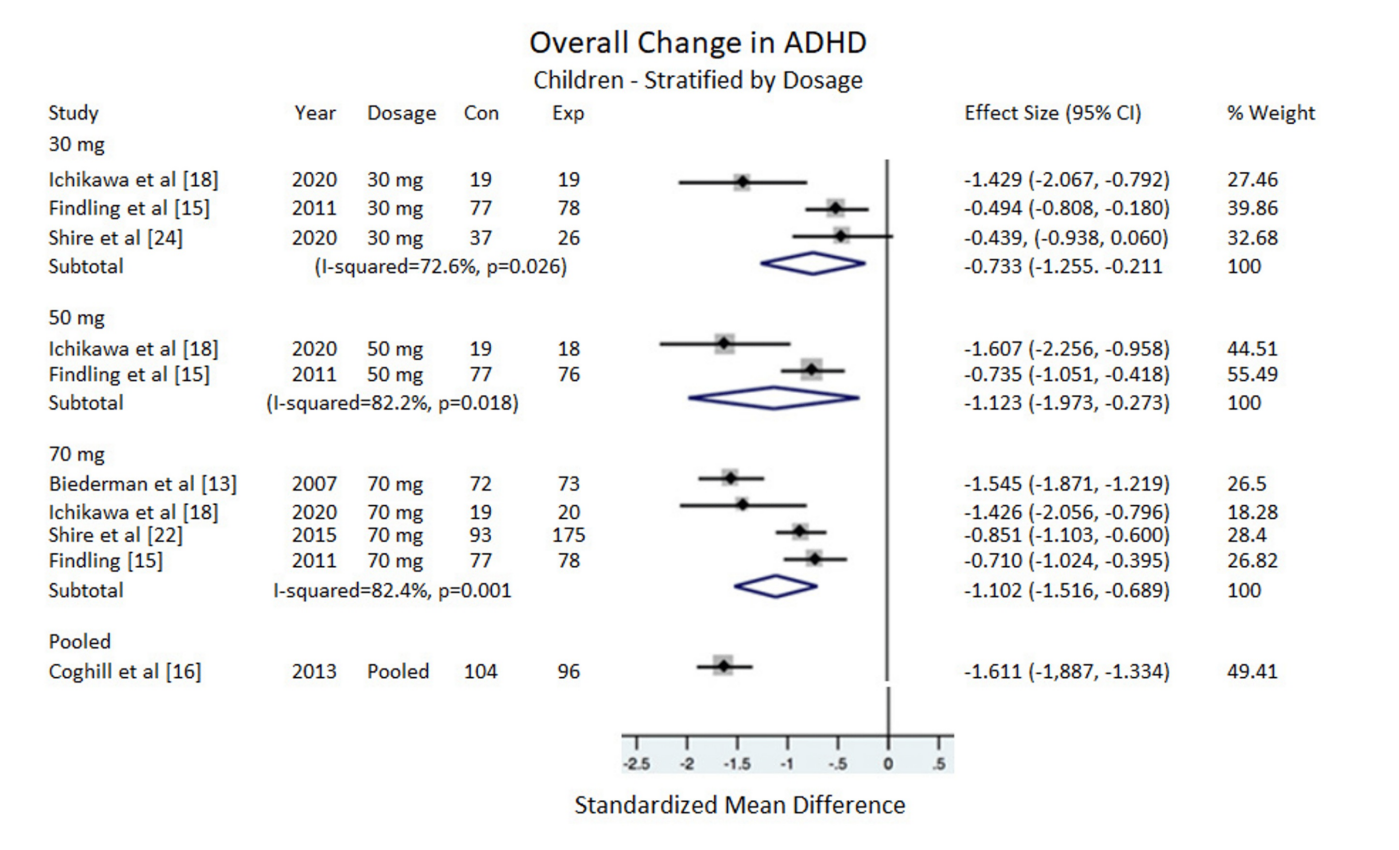
Overall change in ADHD (children) Stratified by dosage. ADHD=Attention deficit/hyperactivity disorder, Con=Control group sample size, Exp=Experimental group sample size.

In children: There was no significant difference between changes in the overall ADHD-RS-IV scale for children based on dosage. The 5 mg, 10 mg, and 20 mg dosages were included in the subtotal displayed in Figure 2, but because these dosages came from only one study, data from these dosages were not included in the pooled analysis displayed in Figure 3. For the 30 mg dosage, ES = -0.733, 95% CI = -1.255 to -0.211, I^2^ = 72.6%. For the 50 mg, ES = -1.123, 95% CI = -1.973 to -0.273, I^2^ = 82.2%. For 70 mg, ES = -1.102, 95% CI = -1.516 to -0.689, I^2^ = 82.4%. For the pooled dosages (30 mg, 50 mg, 70 mg combined), ES = -1.353, 95% CI = -1.853 to -0.852, I^2^ = 85.9% (Figure 3).

Hyperactivity

**Figure 4:**
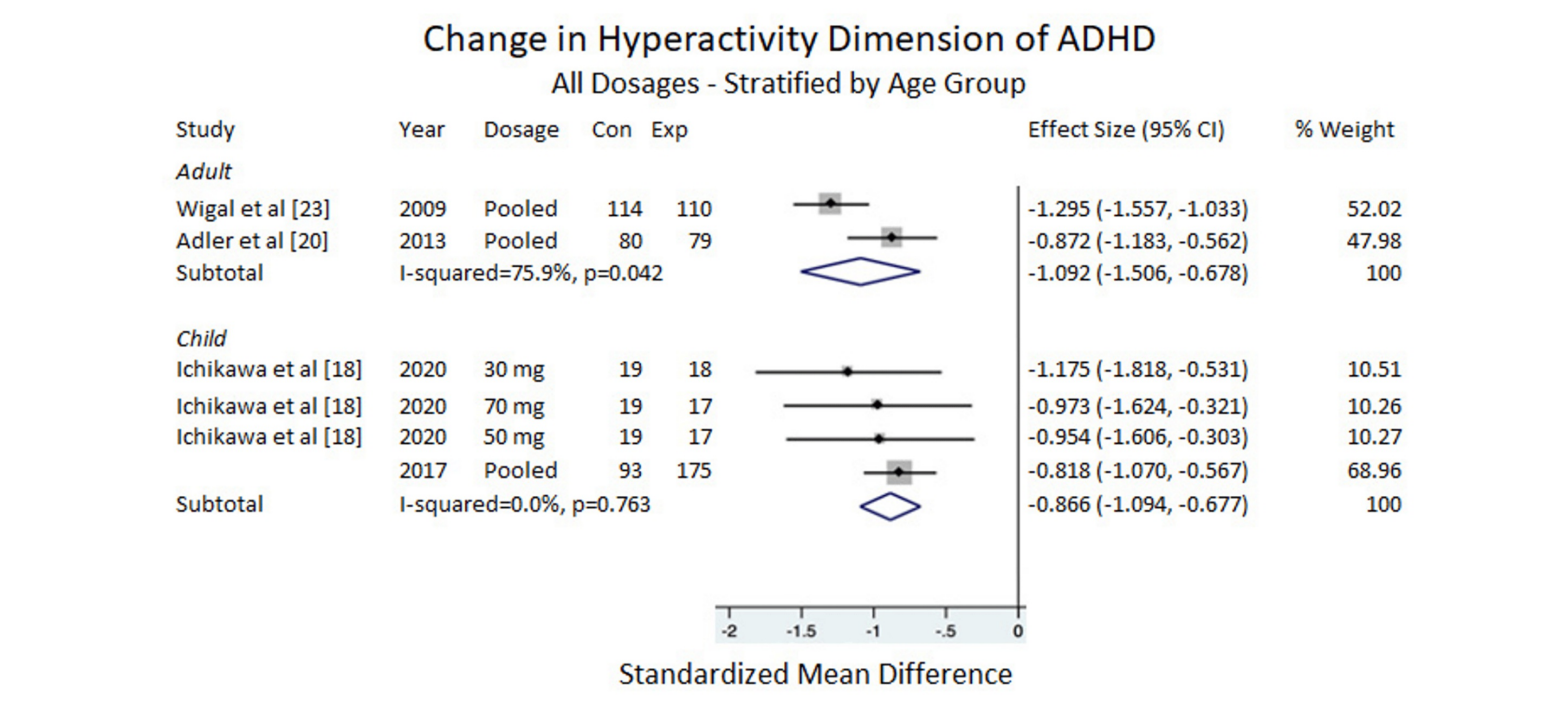
Change in hyperactivity dimension of ADHD All dosages stratified by age group [18,20,23] ADHD=Attention deficit/hyperactivity disorder, Con=Control group sample size; Exp=Experimental group sample size.

Regarding the ADHD-RS-IV subscales, there was a significant clinical improvement for adults, ES of -1.092, 95% CI = -1.506 to -0.678, I^2^ =75.9%. Five items were included in the analysis for children, and the data showed that the difference between the placebo and LDX was significant for clinical improvement, ES = -0.886, 95% CI = -1.094 to -0.677, I^2 ^= 0.0% (Figure 4).

Inattention

**Figure 5:**
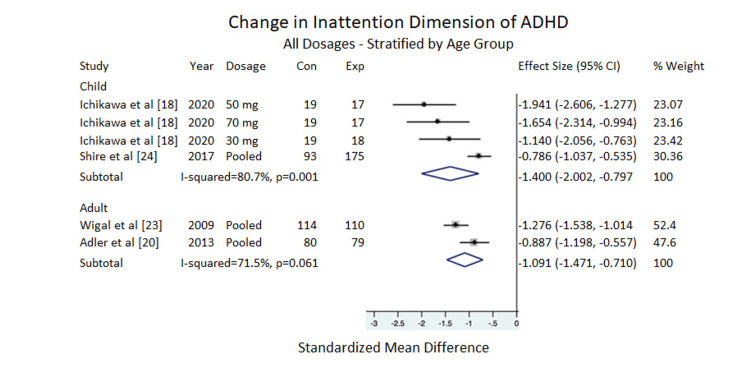
Change in inattention dimension of ADHD All dosages stratified by age group. ADHD=Attention deficit/hyperactivity disorder, Con =Control Group Sample Size, Exp = Experimental Group Sample Size. Note: Weights are from random effects.

Overall, the inattention subscale of the ADHD-RS-IV showed a significant positive difference for adults, ES = -1.091, 95% CI = -1.471 to -0.710, I^2^ =71.5%. For children, ES = -1.400, 95% CI = -2.002 to -0.797, I^2^ = 80.7%, also indicating clinical improvement (Figure 5).

Adverse Events

**Figure 6:**
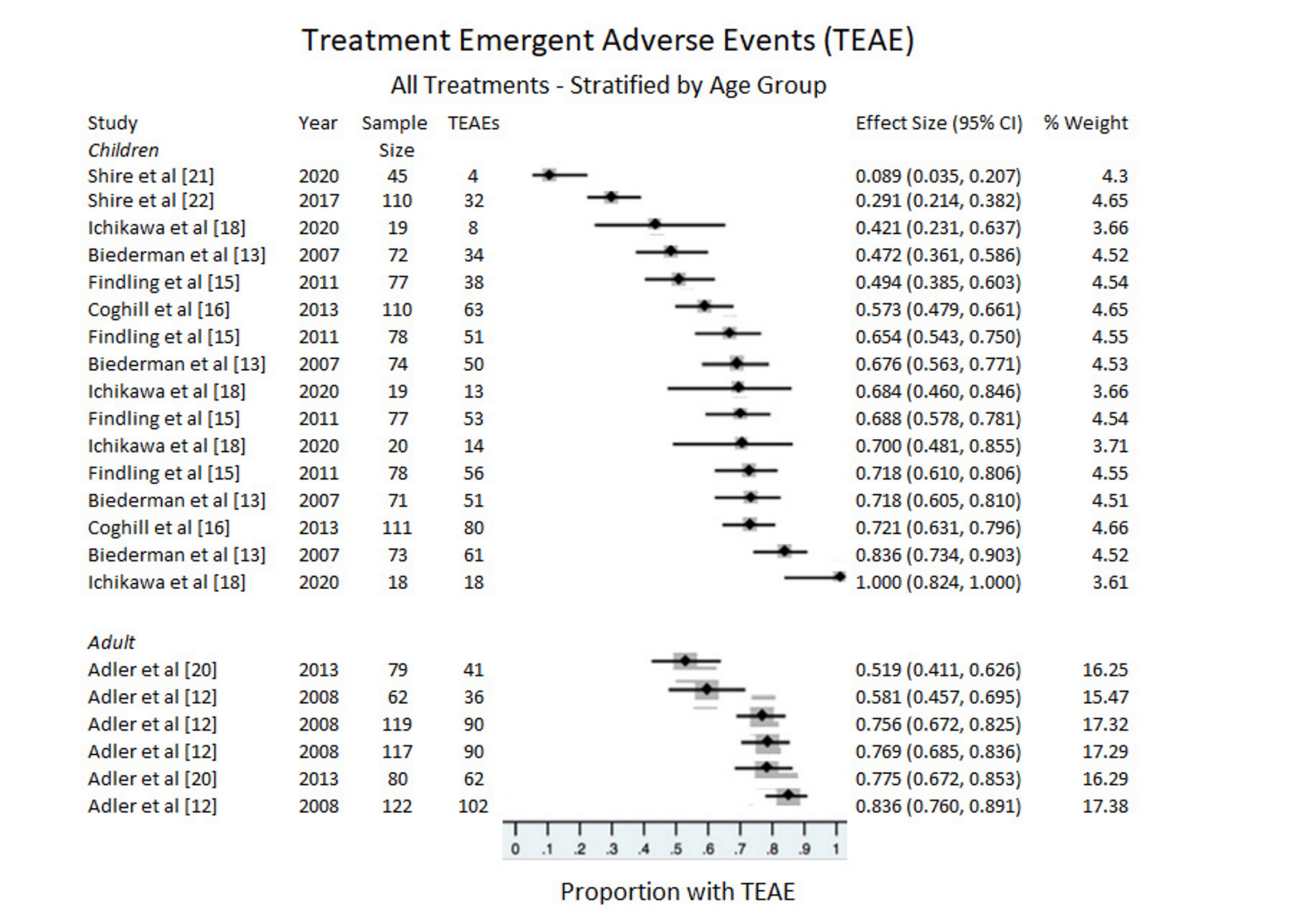
Treatment-emergent adverse events (TEAE) Stratified by age group.

All treatments: Adverse events were measured for both subgroups (adults and children) with no difference between groups (Figure 6). For adults, the proportion with treatment emergent adverse events (TEAE) was, ES = 0.716, 95% CI = 0.618 to 0.805, p for heterogeneity < 0.001, I^2^ = 84.127%. For children TEAE was, ES = 0.564, 95% CI = 0.479 to 0.648), p for heterogeneity < 0.001, I^2^ = 91.460%.

Children Stratified by Dosage

Adverse events were further stratified by placebo and dosage within the child studies (Table 5). For the placebo, the proportion with TEAE, ES= 0.4266, 95% CI= 0.324 to 0.530, p for heterogeneity= 0.000, I^2^= 85.852%. For the 30 mg, ES= 0.671, 95% CI= 0.578 to 0.758, p for heterogeneity= 0.030, I^2^= 62.552. For the 50 mg, ES=0.787, 95% CI= 0.656 to 0.894, p for heterogeneity=0.001, I^2^ = 81.434%. For the 70 mg, ES=0.728, 95% CI= 0.569 to 0.862, p for heterogeneity= 0.000, I^2^ = 91.965%. For the pooled results, ES= 0.750, 95% CI= 0.704 to 0.793. Adverse effects were less common with placebo than LDX.

Stratification of New Dosages

NCT03260205 is the first study to utilize different dosages (5, 10, and 20 mg) for its participants as compared to the standard 30 mg, 50 mg, and 70 mg dosages utilized in the other ten studies [21]. The participants were from a younger age demographic (4-5 years old), so lower dosages were administered. The 5, 10, and 20 mg dosages were included for overall effect measurement. However, the novelty of these dosages in LDX studies made stratification impossible due to a lack of comparable studies. Interestingly, this was the only study that did not find a significant effect with its 30 mg dosage.

Demographics

Race: Only three of the 11 studies reported a racial breakdown [13,15,21]. Though the racial breakdown for the remaining studies was not reported, the other eight included participants from numerous countries, such as the United States, France, Hungary, Spain, Poland, Belgium, the Netherlands, the UK, Italy, Sweden, Canada, and Japan, giving the most diverse sample to date with regards to LDX [13,16-20,22,23].

Sex: Ten of the 11 studies included for the analysis reported a sex breakdown [19]. Eight of these studies included over 62% of males [13,16-18,21-23,26]. This finding is consistent with prior studies that suggest that ADHD is more prevalent in males than females [27].

Age: Although previous meta-analyses investigated both children and adults, our study spans the most extensive age range (4-55 years of age). The Shire et al. (2020) [21] study had the youngest participant sample, which included 4-5-year-olds. The highest mean ages of all 11 studies were observed in Adler et al. (2008) [12] and Adler et al. (2013) [20], with values of 34-35 years.

Discussion

The purpose of this meta-analysis was to assess the efficacy of lisdexamfetamine dimesylate in the treatment of ADHD, focusing on the total ADHD-RS-IV scale and its subscales: inattentiveness and hyperactivity. We further partitioned the analysis by examining the efficacy of varying dosages and subpopulations, including children and adults. Our study’s principal finding is that lisdexamfetamine shows significant efficacy in dosages of 20 mg and above compared to a placebo, similar to previous meta-analyses and systematic reviews [5,14]. While all demonstrate efficacy in their treatment of ADHD, none of the dosages exhibit significance over another. The pooled data demonstrates a higher ES than all individual dosages. While not statistically significant, it does support claims that tailoring individual treatment with particular dosages is crucial in therapy involving LDX [15,28].

The main finding from conducting this meta-analysis was that while specific dosages suggested more substantial effects, there was no significance between the conventional dosages (30, 50, 70 mg). For example, in children, the ES for the 70 mg group is -1.102 while the ES for the 30 mg group was -0.733 (however, this does not reach statistical significance). In adults, the ES for the 70 mg group is -0.904 whereas the ES for the 30 mg group is -0.703, a difference which also did not reach statistical significance. This finding deviates from previous studies that concluded particular dosages of lisdexamfetamine are more clinically significant than others. For example, Biederman found the Least Square Means (LSM) between the 30 mg and 70 mg treatment groups to be -4.91 with p<0.05 [13]. Moreover, Adler found that the 70 mg was also more significant than the 30 mg group in weeks 3 and 4 of their trials [12]. After data aggregation, it appears that while 70 mg does have a higher ES than the 30 mg, a clinically significance between either one is unable to be detected.

When analyzing adverse events, we did not uncover a significant difference between adult and child studies. Pooled results are similar to other amphetamine formulations. Furthermore, there were no significant differences between the different dosages concerning safety. The adverse events showed a potential for no CI overlap between the children and adults. However, the Ichikawa et al. (2020) [18] study introduced high proportionality counts (with the 50 mg dosage showing 100% adverse events). Perhaps with future studies, these results can be partitioned to investigate whether particular dosages or cohorts of participants exhibit varying levels of adverse events.

Clinical Significance

Our work has implications in the medical approach to ADHD and its subsequent treatment with LDX. To our knowledge, there have not been previous meta-analyses that considered both children and adults while analyzing the subscales of the ADHD-RS-IV scale, nor has there been an investigation of the effects of different dosages on treating the symptomatology of ADHD. Our study demonstrated that converse to what previous studies have suggested, there was no significance between doses nor significance between age groups (adults vs. children). For example, both Biederman et al. (2007) [13] and Adler et al. (2008) [12] demonstrated that the 70 mg dosage of LDX was more effective at treating ADHD than the 30 mg LDX. Moreover, Madaan suggested higher doses of LDX would cause a more significant change in severe ADHD as laid out by the ADHD-RS-IV scale [28] We hypothesize that our data aggregation accounts for the lack of major significance between any dosages. Previous studies have also demonstrated that even though LDX was initially developed to treat ADHD in children, it shows efficacy of treating the symptoms of ADHD in adults [4,15,16]. According to previous data and the analytical findings of our study, physicians should consider carefully titrating the dosage to each individual's standards, following standard protocol when changing that dosage.

Analysis of the dosages in the NCT03260205 trial [21] of preschool patients did not show a significant difference between the 30 mg group and other trials in both children and adults that also utilized the 30 mg group. Neither did the 30 mg dose have efficacy compared to placebo. While clinicians often start prescribing LDX at a lower dosage, it appears as though the 30 mg dosage is tolerated quite well. Also, when investigating the adverse events present within the studies, there were no significant differences between both the age cohorts and the individual dosage cohorts, despite there being statistical anomalies (Ichikawa et al. (2020) and its 50 mg group [18]).

There are certain aspects of treating ADHD that this review brings to life. For example, while individual studies did report the mean changes, standard errors, and subscale changes, many did not (although they report the effect sizes or p-values for the overall cohort), making it challenging to analyze the efficacy of ADHD treatments when pooling results. Researchers lose the ability to pool results from an arbitrary scale (i.e., the ADHD-RS-IV scale), a valid measurement of ADHD symptomatology [1,8,11]. Furthermore, specific considerations must be considered when pooling the results of both children and adults together. The adult scale for ADHD-RS-IV allows individuals to self-report their symptoms, while children must have a guardian, parent, or teacher score the symptoms. Finally, recent publications demonstrating differences in the prevalence of ADHD in different demographics, such as race, sex, and socioeconomic status, provide impetus for more research into these realms to see if treatments for ADHD, such as lisdexamfetamine, are comparable between cohorts [29].

Strengths and limitations

Our meta-analysis is the first to incorporate multiple countries into a pooled result for the efficacy of LDX (Japan, European countries, and North American countries). Next, our meta-analysis includes the most studies to date with LDX in both children and adult meta-analyses, seven and four, respectively. Our study is the first to stratify the dosages for LDX and interpret effect measurements for individual dosages in the total ADHD-RS-IV and the respective subscales (inattentiveness and hyperactivity/impulsivity). Finally, our meta-analysis includes the most expansive age range from any previous meta-analysis on LDX (4-5, 6-17, and 18-55 year olds).

There are several limitations associated with this study. While this is the largest study to date on lisdexamfetamine, the sample size is still relatively small. Only a few studies reported the ADHD-RS-IV scale's baseline and endpoints and the differences between the baseline and the endpoint. Another issue was the inability to collect data on the differences in treatment between males and females. Previous studies have demonstrated several instances of a difference in efficacy between males and females [29,30]. This increases the need for more reporting on the sex differences in the scales and their respective subscales when publishing data. Another limitation is that conclusions cannot be drawn for the specific smaller dosages (5, 10, 20 mg) due to the limited use of the NCT03260205 study [21].

Moreover, due to the small sample size presented in individual studies, the statistical power is limited, as the Ichikawa etal. study demonstrated 100% of adverse events in the 50 mg group [18]. The final issue with this meta-analysis was the high heterogeneity of the studies. Due to the already small sample size, high levels of heterogeneity may mitigate the detection of the psychostimulant's efficacy in the treatment of ADHD. However, a random-effects model was conducted to analyze the pooled data and then analyze the individual dosages from each study, which should increase the generalizability of the current study.

## Conclusions

A meta-analysis and systematic review were conducted in May 2024, and at this time, our work is the first meta-analysis that splits the dosages of lisdexamfetamine as well as analyzes the inattentiveness and hyperactivity subscales of the ADHD-RS-IV. We showed that LDX was effective at treating the symptoms of ADHD. Moreover, we were able to separate the dosages and the ADHD-RS-IV subscales to show there was no difference in efficacy, nor were there differences in the safety of LDX. To our knowledge, no previous meta-analysis has approached the subscales and the dosages in this unique way. Future studies need to separate outcome measures not only on dosage but also on sex, age, and race to ensure that all populations are adequately assessed and that no generalizations are made.
